# Understanding patient and parent/caregiver perceptions on gene therapy in Gaucher disease: an international survey

**DOI:** 10.1186/s13023-022-02576-3

**Published:** 2023-01-07

**Authors:** Tanya Collin-Histed, Aviva Rosenberg, Noortje Hopman, Jessica Pacey

**Affiliations:** 1 International Gaucher Alliance, 8 Silver St, Dursley, GL11 4ND UK; 2 67health, 18 Crucifix Ln, London, SE1 3JW UK

**Keywords:** Gaucher disease, Patient survey, Gene therapy, Lysosomal storage disease

## Abstract

**Background:**

Gaucher disease is a rare, autosomal recessive genetic disorder. It is caused by a lack of sufficient activity of the lysosomal enzyme known as glucocerebrosidase, which leads to an accumulation of glucocerebroside, a fatty substance, in the spleen, liver, bone marrow, and rarely, the lungs or central nervous system. While there are several treatments available for people with Type 1 Gaucher disease and the visceral aspects of Type 3 Gaucher disease, no cure is present for any type of Gaucher disease. Clinical trials are currently underway to investigate the safety and efficacy of gene therapy in Gaucher disease, which has the potential to become a new type of (curative) treatment in the future. Gene therapy is a relatively new therapeutic approach, and with the desire to keep the community informed about new treatment developments, the International Gaucher Alliance (IGA) set-up a Gaucher disease specific survey to gauge current perceptions. The survey aimed to benchmark understanding of, and the educational needs surrounding, gene therapy among the Gaucher disease community.

**Body:**

An international, online survey was developed, comprising twelve questions ranging from multiple choice, Likert scale, single tick-box, ranking and open questions. The survey was developed following three patient and caregiver focus groups and underwent review from members of the IGA for readability and accuracy before going live to respondents. The survey was available for two months and shared to audiences via specific Gaucher community channels.

**Conclusion:**

Over 100 patients and parents/caregivers from the Gaucher disease community completed the survey, including people living with Type 1 Gaucher disease (52.88%), people living with Type 3 Gaucher disease (3.85%), parents/caregivers of people living with Type 1, 2 or 3 Gaucher disease (39.42%), and other (3.85%) who were defined as parents of multiple people with Gaucher disease. The survey uncovered various commonalities in perception of gene therapy among all groups, with large knowledge gaps identified on the mode of action, the usefulness of gene therapy and overall understanding of the therapeutic area. This survey provides an overview of the type of information that could be valuable to the Gaucher disease community when developing educational materials.

**Supplementary Information:**

The online version contains supplementary material available at 10.1186/s13023-022-02576-3.

## Background

Gaucher disease is a rare, autosomal recessive genetic disorder. It is caused by a lack of sufficient activity of the lysosomal enzyme known as glucocerebrosidase, which leads to an accumulation of glucocerebroside, a fatty substance, in the spleen, liver, bone marrow, and rarely, the lungs or central nervous system [1, 2]. Gaucher disease takes three main forms: Type 1, 2, and 3, which vary widely in clinical manifestations. In Europe, Israel, Canada, and the USA, Type 1 is the most prevalent form (94% of cases); in other countries, such as Egypt, India, Japan, Poland and Sweden, neuronopathic forms (Types 2 and 3) can be more prevalent than Type 1 [3]. It is thought that Type 1 Gaucher disease affects 1 in 100,000 people in the general population, and 1 in 850 people of Ashkenazi Jewish descent [2]. Symptoms of Gaucher disease can appear at any age, from infancy through to adulthood, and include anemia, fatigue, easy bruising, and a tendency to bleed. An enlarged spleen and liver with a protruding stomach may also occur as well as bone pain, loss of bone strength and density with an increased risk of fractures. In cases of neuronopathic Gaucher disease (Types 2 and 3) neurological symptoms such as eye movement disorder, unsteadiness, seizures and cognitive decline can be present. Type 2 Gaucher disease typically affects infants and is apparent by 6 months of age. Infants may appear healthy at birth but develop neurological symptoms by the age of 3–6 months and typically die in infancy [3]. Children with Type 3 Gaucher disease may present with similar although more severe symptoms as Type 1, but with time, develop neurological signs, such as cognitive impairment, myoclonic seizures, ataxia, spasticity, horizontal saccade initiation failure, incomplete vertical gaze, abnormally slow object tracking, and convergent squint and muscle weakness [3].

Current treatment for Gaucher disease focuses on Enzyme Replacement Therapy (ERT) to improve symptoms associated with the disease, for example, enlarged spleen and liver, low red blood cell count and bone pain [4], and Substrate Reduction Therapy (SRT), to reduce the number of fatty substances in the cells of people with Gaucher disease [5]. There is currently no cure for Gaucher disease. Over the last few years, researchers have begun investigating gene therapy as a potential new treatment option for people living with Gaucher disease. Gene therapy modifies a person’s genes to treat or cure a disease, it does this in a multitude of ways, by replacing a disease-causing gene with a healthy copy of the gene, inactivating a disease-causing gene that is not functioning properly and introducing a new or modified gene into the body to help treat a disease [6]. With four gene therapies approved by the Food and Drug Administration (FDA) to date for several other disease conditions [7] and ongoing clinical trials by multiple pharmaceutical and biotechnology companies investigating the safety and efficacy of gene therapy in Gaucher disease specifically [8], this therapeutic option may become a new type of (curative) treatment in the future. However, to help current and future clinical trials work with the community and patient and family expectations, perceptions of gene therapy must be understood, and educational tools put in place to tackle any uncertainty arising from those perceptions. The aim of this article is to explore the educational needs identified by the Gaucher disease community when learning about gene therapy.

## Main text

### Introduction

To understand the level of knowledge around gene therapy and perceptions of the Gaucher disease community, the IGA engaged people living with Gaucher disease and parents/caregivers through qualitative (virtual focus groups) and quantitative (online survey) methods. As a first step, the IGA held three 90-min virtual focus group discussions with members of the Gaucher disease community from around the world. Each group included 5–7 members, comprising people living with Type 1 Gaucher disease, parents/caregivers of people living with Type 2 and Type 3 Gaucher disease and young adults living with Type 3 Gaucher disease. Each group was asked a set of seven guided questions. These questions resulted in several major recurring themes, one of which was better understanding of gene therapy and the desire to be further educated on new treatments. While generating themes from the focus groups was valuable, the sample size was limited, prompting the need for a broader, larger group of respondents to gauge whether the wider community had aligned opinions and understanding of this relatively new therapy.

### Survey design and development

Via the survey website, SurveyMonkey, an online questionnaire was set-up to gauge the understanding and educational needs of the Gaucher disease community. The survey assessed the opinions, needs, and wishes of the Gaucher disease community via a questionnaire comprising 12 questions spanning multiple choice, Likert scale, single tick-box, ranking and open questions. The basis for these questions originated from prior qualitative research during the focus groups and subsequent, collaborative discussions with industry partners following the presentation of the outcomes of said focus groups. Ahead of disseminating the survey online, the 12 questions underwent review by members of the IGA for readability and accuracy. Personal identification questions were not asked to ensure privacy and anonymity of the respondents. The questions were designed to investigate overall knowledge of gene therapy, specific topics associated with gene therapy and personal concerns and hopes of each individual when thinking about gene therapy. Each question was developed with the aim to identify different levels of understanding and to determine the topics that future educational materials should focus on.

Following set-up of the survey and quality assurance, the survey hyperlink was shared with members of the IGA—asking them to disseminate it to their individual networks. Over two months, the IGA’s target of 100 responses was achieved, resulting in the survey being closed and data analyzed.

### Participants

The international, online survey comprised 104 respondents from the Gaucher disease community. Survey participants were either people living with Type 1 or Type 3 Gaucher disease or parents/caregivers of people living with Gaucher disease Type 1, 2 or 3. In total, 29 countries across five continents were represented; for an overview, please see Table 1. The highest number of respondents originated from the United States (33) and the United Kingdom (13), which could be a result of the survey questions and communication being in English language only. The average age group was 35–54 years (Table 2), which accounted for almost half of the respondents. Survey respondents were not asked to stipulate their gender or disclose any personally identifiable information.

**Table 1:** Overview of countries/continents and number of respondents

Continent	Country	Number of respondents
North American	United States of America	33
	Canada	3
South America	Mexico	2
	Peru	1
	Argentina	1
	Paraguay	1
	Guatemala	1
Asia	Pakistan	5
	India	3
	Japan	3
	China	2
	Thailand	1
	Israel	1
Europe	United Kingdom	13
	Slovenia	8
	Denmark	4
	Sweden	4
	Italy	3
	Croatia	2
	Romania	2
	Greece	2
	France	1
	Lithuania	1
	Bosna and Herzegovina	1
	Norway	1
	Portugal	1
	North Macedonia	1
	Poland	1
Africa	Kenya	1
Unspecified	Unspecified	1

**Table 2:** Overview of age groups

What is your age?	Responses (%)	Responses (*n*)
Under 18	6.73%	7
18–24	2.88%	3
25–34	13.46%	14
35–44	25.00%	26
45–54	24.04%	25
55–64	14.42%	15
65 +	13.46%	14

### Results

Using a scaling question, respondents were first asked about their understanding of gene therapy. Of the respondents, 9.62% indicated they did not understand gene therapy, 35.58% indicated they had very little knowledge but are learning and 11.54% indicated they were experts in gene therapy. Results showed the remainder of respondents were in-between categories, having certain knowledge about gene therapy but not viewing themselves as fully educated on the topic.

Following the initial question on general understanding of gene therapy, the next question asked about specific topics within gene therapy, e.g., clinical trials, safety of the treatment, and respondents were asked to rank their level of knowledge on these topics. Respondents stated they were well informed about ‘availability of gene therapy’ and ‘clinical trials/studies in gene therapy’, while the topics ‘long-term implications’ and ‘preparing for treatment’ were ranked low.

The next question was an open question in which respondents were asked to explain what first came to mind when thinking about gene therapy. Answers included whether it would be a game-changer, how effective it is, what the risks are and its mode of action. Other responses were less treatment specific and questioned the price and the current political landscape (Additional file 1). Once respondent’s initial thoughts were collected, they were asked about their hopes for gene therapy within the Gaucher disease community. More than three-quarters of respondents (75.73%) mentioned the potential for a cure, and as multiple choices were recommended for this question, both ‘opportunity to maintain or improve current lifestyle’ (73.73%) and ‘slow or stop disease progression’ (71.84%) rated highly among respondents.

When asked about the main barriers against considering gene therapy, through a second multiple choice question, answers centered around the long-term implications being unknown (21.57%), the respondent not being educated enough on gene therapy to make an informed decision (19.61%) or the risks and benefits being unclear (18.63%). Emotionally loaded multiple choice options had little engagement, with ‘I don’t want to think about it right now’ and the ‘I fear gene therapy may make my condition worse’ ranking the lowest with respondents, 1.96% and 2.94% respectively.

Beyond questions on gene therapy itself, respondents were asked questions on the types of materials they would find useful when learning about gene therapy and where the materials should be hosted. While many different types of materials and resources (e.g., face-to-face, print, online) were selected as valuable, webinars, live meetings and informational websites were favored by the respondents (with a minimum of 59% preference). In terms of hosting location for the materials, the respondents ranked informational websites and patient group resource centers equally the highest at 66.35%. The penultimate question asked about clinical trials, to establish what information a person living with Gaucher disease would need to make an informed decision about participating in a clinical trial. The responses focused on objective measures, including seeing the results of previous clinical trials (73.08%), understanding possible outcomes (68.27%), understanding what is involved in the procedure itself (66.35%) and overview of the potential side effects and risks (66.35%).

Finally, in an open question, respondents provided a variety of responses when asked what they would like to see happen in gene therapy for Gaucher disease. Many reiterated the need for educational materials, news about treatments and updates on clinical effectiveness and side effects. Others discussed the hope for improved quality of life, a potential cure, and a stop to invasive treatments.

## Conclusions

In conclusion, the survey data suggests a need for educational materials on gene therapy in Gaucher disease. While there are commonalities amongst people living with the disease and their parents/caregivers in terms of information required, e.g., treatment side effects, long-term data, and effectiveness, there are also disparities in terms of the level of knowledge about gene therapy. Due to these knowledge gaps and to ensure materials meet the needs of all audiences interested in gene therapy in Gaucher disease, it would be recommended that:

- Educational materials are developed across a variety of topics—to cover multiple areas of interest
- Educational materials are tiered as ‘foundational’ and ‘advanced’—to satisfy different knowledge levels
- Educational materials are available primarily on informational websites and via patient group resource centers in the form of webinars, live meetings, and online tools

When analyzing the feedback from respondents, three key topics emerged and should be considered when developing educational materials:

- Medical (effectiveness, risks, and side effects of gene therapy)
- Prognosis (long term implications of gene therapy and impact on quality of life for people living with Gaucher disease)
- General understanding about gene therapy and clinical trials (how gene therapy works, the treatment process and clinical trials that are available)

The results of this international survey are a steppingstone to better understand the perceptions, beliefs and educational needs of people living with Gaucher disease and their parents/caregivers when considering and learning about gene therapy. These insights will help associations like the IGA and industry groups develop informative and relevant educational materials for the Gaucher disease community to increase awareness and knowledge of gene therapy and keep them informed about the latest developments in this therapy area.

## Supplementary Information

**Additional file 1.** Collection of respondents' initial thoughts on gene therapy.

## Data Availability

The dataset used and/or analyzed during the current manuscript are available from the authors on reasonable request.

## Abbreviations

IGA: International Gaucher Alliance
GT: Gene therapy
