# Supplementary material for: Understanding patient and parent/caregiver perceptions on gene therapy in Gaucher disease: an international survey
Source: Orphanet J Rare Dis. 2023 Jan 7;18:5. doi: 10.1186/s13023-022-02576-3 (PMC9824927; doi:10.1186/s13023-022-02576-3)
Supplement: Supplementary file 1 — Additional file 1. Collection of respondents' initial thoughts on gene therapy. [file 13023_2022_2576_MOESM1_ESM.docx]

Survey data question 06: Collection of respondents’ initial thoughts on gene therapy

| **When you think of gene therapy, what is first thought or question that comes to mind?** | |
| --- | --- |
| Medical | Safety and reliability |
|  | Safety of my home - because my father impliments me it |
|  | Effectiveness |
|  | Safety, and ability to replace current treatment. Cure? |
|  | How effective is it |
|  | What are the risks! |
|  | Normal ERT without complications |
|  | Hope, but also concerns regarding risk |
|  | Extremely risky, but with very good benefits to counteract the risks |
|  | Is it life savings or not |
|  | The safety of the therapy |
|  | How effective is it expected to be? |
|  | Success rate |
|  | Is it safe |
|  | Single treatment |
|  | How safe is it? |
|  | Safety of treatment not yet acceptable compared to other available therapies |
|  | What is the risk of having the treatment |
|  | Is it safe? |
|  | What are the risks? |
|  | Is this a safe treatment? |
|  | Intrusiveness of procedures |
|  | Safety of the treatment |
|  | Safety |
|  | Efficacy, availability, cost |
|  | Safety |
|  | Safety |
|  | Safety |
|  | It is safe? |
|  | How safety and effectiveness of the treatment |
| Prognosis | Gene therapy help patients to overcome or slow down the cause disease. |
|  | Will it work for type 3 patients |
|  | Long term solution |
|  | Will this be a long-term solution or cure? |
|  | Better quality of life |
|  | No more IV because you get a functional copy of gene. |
|  | At what age does the cost benefit move against its use |
|  | What is the success rate |
|  | Definitive cure for the Gaucher disease\| |
|  | Will it succeed? |
|  | Will it be the last treatment I receive about my disease until the end of my life? Will there be long-term side effects? |
|  | Hope and uncertainty. |
|  | Better lifestyle |
|  | Will it truly be a one and done? |
|  | Will it make your life better or worse? |
|  | Long term |
|  | will it truly be a one-off treatment |
|  | Will it be a game-changer or not? I mean, will it be a chance for patients to be completely healthy? |
| General understanding about gene therapy and clinical trials | If it’s better than enzymatic therapy |
|  | How it works? |
|  | When will be possible to stop treatment for life? |
|  | How affordable will it be? |
|  | Genetic alteration to generate missing enzymes |
|  | How to implement it into Gaucher disease. When and where does the clinical trial for GD start? What is the risk and side effect? |
|  | Bone marrow |
|  | Cure! |
|  | Ali bom imela manj težav ki so posledica bolezni |
|  | who gene |
|  | Cure |
|  | Is it a permanent cure? |
|  | The cure. |
|  | When it can be used for Gaucher type 3 disease |
|  | Can I get it |
|  | How it works? |
|  | A cure |
|  | Will it cure GD completely? |
|  | When will it be available to me? |
|  | Or having to keep doing infusions |
|  | Replacing a defective mutation or gene with a new one that is not defective |
|  | Medical breakthrough technology |
|  | Curing diseases |
|  | I feel hope |
|  | conditioning (chemotherapy) |
|  | fixing my Gaucher disease |
|  | If something seems too good to be true, it may not be. The Titanic could have used the lifeboats that the best minds thought were unnecessary. |
|  | Introduce new gene to the cell |
|  | Science fiction. Expensive. Back to normal. |
|  | A cure! |
|  | Scientific experiments that will hopefully one day become an effective treatment for Gaucher. |
|  | New, unknown |
|  | Playing around with our genes |
|  | How does it work? |
|  | A bit scary (new, experimental, invasive) but exciting (innovative, cure) |
|  | at last, we have arrived at gene therapy awaiting for many years. |
|  | Will it work? |
|  | Thank God!!!! It’s changed my life. |
|  | IF IAM GOING TO BE CURED COMPLETELY |
|  | Stem cell |
|  | Is there going to be gene therapy for Gaucher Disease? |
|  | does it work |
|  | How complicated is it, does it work and will insurance pay for it? |
|  | The first thought is slides and pipettes |
|  | I hope it will soon be possible to use gene therapy I a safe way, but sometimes I fear that political discussion can stop or delay the progress. |
|  | It‘s like a light full of hope when I have no choice, perhaps can change my child's life. |
|  | A cure!! Wow!! |
